# Phenotypic landscape of non-conventional yeast species for different stress tolerance traits desirable in bioethanol fermentation

**DOI:** 10.1186/s13068-017-0899-5

**Published:** 2017-09-13

**Authors:** Vaskar Mukherjee, Dorota Radecka, Guido Aerts, Kevin J. Verstrepen, Bart Lievens, Johan M. Thevelein

**Affiliations:** 1 Laboratory of Molecular Cell Biology, Institute of Botany and Microbiology, VIB Center of Microbiology, KU Leuven, Kasteelpark Arenberg 31, B-3001 Louvain, Belgium; 2 Laboratory for Enzyme, Fermentation and Brewing Technology (EFBT), Department of Microbial and Molecular Systems, KU Leuven, Technology Campus Ghent, Gebroeders De Smetstraat 1, B-9000 Ghent, Belgium; 3 Laboratory for Process Microbial Ecology and Bioinspirational Management (PME&BIM), Department of Microbial and Molecular Systems, KU Leuven, Campus De Nayer, Fortsesteenweg 30A, B-2860, Sint-Katelijne Waver, Belgium; 4 Laboratory for Systems Biology, VIB Center for Microbiology, KU Leuven, Gaston Geenslaan 1, B-3001 Louvain, Belgium; 5 Present Address: Lundberg Laboratory, Department of Marine Sciences, University of Gothenburg, Medicinaregatan 9C, 41390 Göteborg, Sweden

**Keywords:** Yeasts, Non-*Saccharomyces*, Phenotype, Fermentation, Stress tolerance, Bioethanol

## Abstract

**Background:**

Non-conventional yeasts present a huge, yet barely exploited, resource of yeast biodiversity for industrial applications. This presents a great opportunity to explore alternative ethanol-fermenting yeasts that are more adapted to some of the stress factors present in the harsh environmental conditions in second-generation (2G) bioethanol fermentation. Extremely tolerant yeast species are interesting candidates to investigate the underlying tolerance mechanisms and to identify genes that when transferred to existing industrial strains could help to design more stress-tolerant cell factories. For this purpose, we performed a high-throughput phenotypic evaluation of a large collection of non-conventional yeast species to identify the tolerance limits of the different yeast species for desirable stress tolerance traits in 2G bioethanol production. Next, 12 multi-tolerant strains were selected and used in fermentations under different stressful conditions. Five strains out of which, showing desirable fermentation characteristics, were then evaluated in small-scale, semi-anaerobic fermentations with lignocellulose hydrolysates.

**Results:**

Our results revealed the phenotypic landscape of many non-conventional yeast species which have not been previously characterized for tolerance to stress conditions relevant for bioethanol production. This has identified for each stress condition evaluated several extremely tolerant non-*Saccharomyces* yeasts. It also revealed multi-tolerance in several yeast species, which makes those species good candidates to investigate the molecular basis of a robust general stress tolerance. The results showed that some non-conventional yeast species have similar or even better fermentation efficiency compared to *S. cerevisiae* in the presence of certain stressful conditions.

**Conclusion:**

Prior to this study, our knowledge on extreme stress-tolerant phenotypes in non-conventional yeasts was limited to only few species. Our work has now revealed in a systematic way the potential of non-*Saccharomyces* species to emerge either as alternative host species or as a source of valuable genetic information for construction of more robust industrial *S. serevisiae* bioethanol production yeasts. Striking examples include yeast species like *Pichia kudriavzevii* and *Wickerhamomyces anomalus* that show very high tolerance to diverse stress factors. This large-scale phenotypic analysis has yielded a detailed database useful as a resource for future studies to understand and benefit from the molecular mechanisms underlying the extreme phenotypes of non-conventional yeast species.

**Electronic supplementary material:**

The online version of this article (doi:10.1186/s13068-017-0899-5) contains supplementary material, which is available to authorized users.

## Background

Association of yeasts with human activity for the production of fermented beverages predates 7000 BC [1]. Yeasts are one of the most extensively studied microbial groups in nature. However, the majority of yeast-related research has been focussed on the ascomycete yeast *Saccharomyces cerevisiae* due to its centuries-long utilization in the production of fermented food and beverages [2–5], its multiple advantages as a model organism, e.g., its small and compact genome [6] and ease of genetic modification. During evolution, *S. cerevisiae* accrued several traits that turned out to be highly beneficial for food and beverage fermentations. Its ability to proliferate regardless of the availability of oxygen [7, 8], tolerate, and accumulate high ethanol concentrations [9] and ability to flocculate [10] are important examples. As a result, over time brewers, winemakers, and other fermented food and beverage producers picked *S. cerevisiae* as the preferred choice for starter cultures [9]. More recently, however, second-generation bioethanol production with yeast has become an important industrial application demanding several other critical traits that are absent in the current industrial *S. cerevisiae* strains. In addition to the requirement for xylose fermentation, it also requires tolerance to the harsh environmental conditions (e.g., osmotic stress, heat stress, ethanol stress) and the presence of multiple inhibitory compounds (e.g., weak acids, furan aldehydes, phenolic compounds) that are typically present in the lignocellulosic hydrolysates used in second-generation bioethanol production [11–14]. As a result, use of an *S. cerevisiae* strain intended for food or beverage fermentations or even for first-generation bioethanol production with starch hydrolysates from corn or wheat is unable to support efficient second-generation bioethanol production. Therefore, current research focuses on exploring the natural diversity of *Saccharomyces* for strains with higher stress and inhibitor tolerance [15] and on developing superior *S. cerevisiae* strains for second-generation bioethanol production using evolutionary engineering and/or targeted genetic modification [16, 17].

Another approach is the use of non-conventional xylose-utilizing yeast species that can produce ethanol under environmentally stressful conditions [18]. Several thousands of non-conventional yeast species have been described and many more are likely to exist [19]. Most of these yeast species have been isolated from fermented or spoiled food and beverages, clinical samples, and environmental samples such as soil and plants [19]. Geographically, a great majority of the available yeast strains is derived from western Europe, Japan, and North America, leaving us with many, large unexplored areas [19]. Interestingly, several non-conventional yeast species show extreme stress tolerance phenotypes, which are unavailable in any natural or industrial *S. cerevisiae* strain [20]. Hence, these unexplored regions may harbor interesting host strains or contain interesting genetic information for the development of superior strains for production of second-generation bioethanol. Non-conventional yeast species are ubiquitous in all sorts of niches, which results in extensive sequence divergence between different lineages of the same species [21]. Therefore, exploring the huge, yet barely exploited diversity of non-conventional yeasts presents an excellent opportunity to achieve the following objectives. First, it allows identification of new extremophile yeast species that can be used to unravel the molecular basis of these extreme phenotypes and transfer the properties to industrial *S. cerevisiae* host strains. Second, it may allow identification of new multi-tolerant ethanol-fermentative yeast strains that could be used as production organism. Since this work was focused on stress tolerance characteristics, pentose fermentation capacity was not evaluated. Conferring xylose fermentation capacity to industrial *S. cerevisiae* strains is now well established [16, 22].

Previously, several species have been identified and characterized in more detail for their tolerance to extreme stress conditions pertinent for second-generation bioethanol production. For example, *Zygosaccharomyces rouxii* is able to grow in media with up to 90% (w/v) of sugar [23], *Kluyveromyces marxianus* is able to ferment efficiently at high temperature [24], *Zygosaccharomyces bailii* is known to tolerate high concentrations of acetic acid along with elevated osmotic pressure [23, 25], and *Pichia kudriavzevii* is able to sustain high furan aldehyde concentrations [26]. There are still many other yeast species that have never been characterized for tolerance to stress factors associated with second-generation bioethanol production. Also, the propagation and phenotyping conditions used in previous studies varied widely.

In this study, we explored under the same standardized conditions the potential of 232 isolates belonging to 82 different non-conventional yeast species for possessing stress tolerance traits important in second-generation bioethanol production. First, strains were subjected to high-throughput phenotypic screening for osmotolerance, ethanol tolerance, thermotolerance, halotolerance, tolerance to furan aldehyde, and tolerance to heavy metal contaminants. Next, the ethanol fermentation potential of a selection of multi-tolerant strains was evaluated in the presence of different stress factors. And finally, the fermentation performance of the most stress-tolerant strains was evaluated in small-scale, semi-anaerobic fermentations with lignocellulose hydrolysate as substrate.

## Methods

### Collection, identification, and storage of yeast strains

In this study, a large non-conventional yeast strain collection was used consisting of 232 non-*Saccharomyces* strains isolated from diverse origins. A large number of these strains were isolated from spoiled or contaminated foods and beverages, such as grape must, cucumber brine, sugar kefir, molasses, cherry, dates, plums and orange juice. Strains originating from spontaneous fermentations such as cacao, lambic beer, fermenting honey, tea beer also comprise a large part of the strain collection. The collection also has strains originating from flowers, nectar, and soil. Details on the isolation and geographic origin of most of the strains are available in the supplementary information (Table S2). All strains used in this study were identified up to the species level using the protocol described previously by Kurtzman and Robnett [27]. Briefly, genomic DNA was extracted using zymolyase treatment of the cells (Seikagaku Biobussiness, Tokyo, Japan). A single colony was dissolved into 50 μL of lysis solution [3 mg zymolyase mL ultrapure water (Millipore, Billerica, MA)^−1^]. The solution was heated at 37 °C for 60 min, followed by 10 min at 98 °C. The variable D1/D2 domain of the large-subunit (26S) rDNA gene was amplified using primers NL-1 and NL-4. The PCR product was purified and sequenced by the VIB Genetic Service Facility (Antwerp, Belgium) using Applied Biosystems 3730XL DNA Analyzer [27]. Identification was performed by BLAST analysis in Gen-Bank. The strains used in this study belong to 81 different non-conventional yeast species. Additionally, nine *Saccharomyces cerevisiae* strains were also included in the study for comparative purposes. All species and the number of strains from each species are mentioned in Table 1. All strains were stored at −80 °C using glycerol-based standard storage medium (Bacto peptone 2% w/v, Yeast extract 1% w/v, Glycerol 25% v/v) in 96-well microtiter plates. Five strains from different origins were present in each microtiter plate as control to estimate the inter-experiment variation.

**Table 1:**
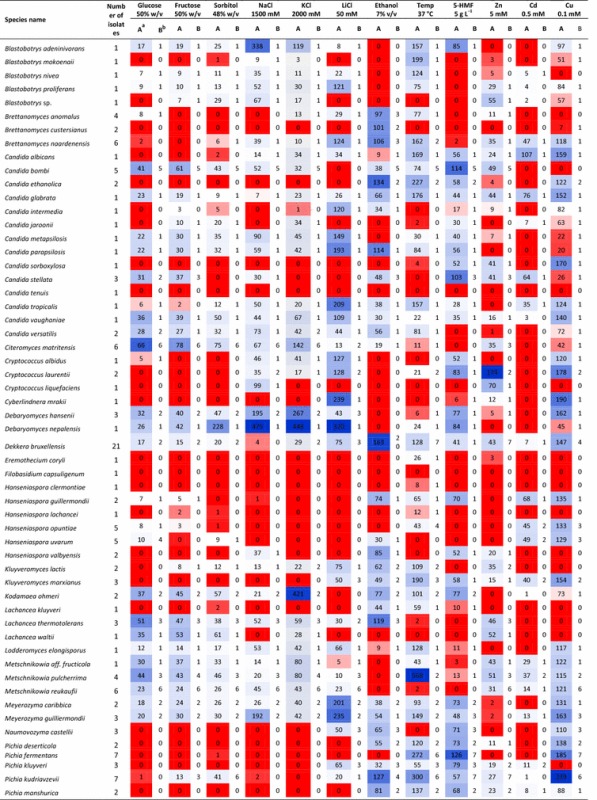

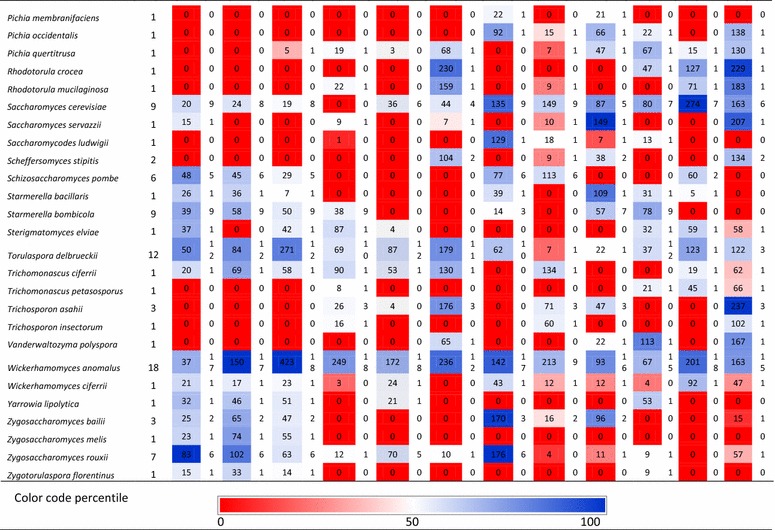
Overview of the performance of non-conventional yeast strains for different traits desirable in second-generation bioethanol production

^a^Column A represents the maximum relative growth attained by a strain of a species at reference condition

^b^Column B represents the number of strains of a species that managed to grow at reference condition with relative growth >5%

### Selection of the test conditions and media preparation

Growth of all strains was evaluated under stress conditions relevant for second-generation bioethanol fermentations. The test conditions were selected as described previously by Mukherjee and co-workers (2014). Unless mentioned otherwise, all chemicals used for media preparation were purchased from Sigma-Aldrich (St. Louis, MO, USA). Osmotolerance was evaluated by using increasing concentrations of glucose (ranging from 40 to 70% w/v), fructose (40–70% w/v), and sorbitol (30–55% w/v). Similarly, halotolerance [NaCl (500–3000 mM), KCl (1000–4000 mM), and LiCl (10–600 mM)]; ethanol tolerance [ethanol (5–15% v/v)]; furan derivative tolerance [5-HMF (2–7 g L^−1^)]; and heavy metal tolerance [ZnCl_2_ (1–10 mM), CuSO_4_ (0.1–2 mM), and CdSO_4_ (0.25–3 mM)] were evaluated using increasing concentrations of the stress factor. Growth assays were performed using yeast extract peptone dextrose (YPD) agar plates [(1.5% w/v agar (Invitrogen, Carlsbad, CA, USA), 2% w/v bacto peptone (Becton–Dickinson, East Rutherford, NJ, USA), 1% w/v yeast extract (Lab M, Heywood, Lancashire, UK), and 2% w/v glucose] supplemented with the test compounds. In order to evaluate thermotolerance, isolates were grown on standard YPD agar (without any test compound) and incubated at 24–41 °C. In addition, isolates were also grown on standard YPD agar plates to evaluate growth in a stress-free environment and considered in this study as control condition [15].

### Phenotypic evaluation and data analysis

All isolates were spotted on different test plates using a high-density array robot (ROTOR HDA, Singer Instruments, Roadwater, Somerset, UK) in order to improve high throughput and reproducibility. The spotting assay was performed following the protocol described in previous studies [15, 28]. Briefly, the isolates stored at −80 °C were thawed, spotted in standard YPD agar plates, and incubated at 30 °C for 48 h. Next, a 96-well plate containing 150 μl of liquid YPD medium (2% w/v bacto peptone; 1% w/v yeast extract and 2% w/v glucose) in each well was inoculated with these freshly grown strains and precultured overnight at 30 °C on a microplate platform shaking at 900 rpm. While screening for ethanol tolerance, yeast strains were preconditioned for 48 h by adding 2% v/v ethanol in liquid YPD preculture medium. After preculture, the growth of each strain was estimated by measuring optical density at 600 nm (OD_600_) using a microplate reader (Molecular Devices, USA) and the cell density was manually adjusted to OD_600_ ≈ 0.2. Finally, this culture was used for spotting on the test plates using the HDA rotor. All test plates were incubated for 5 days at 30 °C (except plates for thermotolerance screening, which were incubated at the indicated test temperature). Growth in each test condition was measured by quantifying the spot size. This was achieved by scanning images of the test plates and analyzing the images using a dedicated image processing software ImageJ [29] combined with the ScreenMill plugin [30]. Next, relative growth (RG) of each strain in a specific test condition was calculated by dividing the actual growth of the strain on that test plate by the growth of that strain on control medium. In this study, growth of a strain in any test condition is considered only when the RG ≥5%. Maximal tolerance limits were defined for each species when at least one strain of that species had an RG ≥5%. In order to compare tolerance of a strain to that of other isolates, a reference concentration for each trait was determined where approximately 50% of the investigated isolates managed to grow (Table 1).

### Fermentation potential of selected multi-tolerant strains

Subsequently, 12 multi-tolerant strains belonging to 12 different yeast species with the best overall performance in high concentrations of glucose, ethanol, 5-HMF, and growth at high temperature were selected and subjected to small-scale semi-anaerobic batch fermentations (100 mL). Two commercial bioethanol producing *S. cerevisiae* strains (Ethanol Red and CAT1) were included in the fermentation assay as references. The selected strains were subjected to fermentation in YP (2% w/v bacto peptone; 1% w/v yeast extract) + 10% w/v glucose medium (further referred to as “YP 10% glucose”) to determine their fermentation potential under stress-free conditions. Additionally, fermentation performance in the presence of different stress factors relevant for second-generation bioethanol production was evaluated separately. This was done by supplementing the fermentation medium YP 10% glucose (w/v) with different stress factors, including 50% w/v sorbitol (very-high-gravity); 0.8% v/v acetic acid (weak acid tolerance), and 3 w/v g L^−1^ 5-HMF (furan aldehyde tolerance). All fermentations were incubated at 30 °C except for the fermentations under heat stress which were incubated at 40 °C. Strains were precultured overnight in 3 mL YPD at 30 °C. Subsequently, strains were grown until stationary phase in 50 mL YPD at 30 °C, 200 rpm for 2 days with a starting OD_600_ of 0.75. Next, OD_600_ of the pre-cultures was measured and 100 mL semi-anaerobic batch fermentations were started in 300-mL schott flasks with a starting OD_600_ of 4 (for very-high-gravity) or 0.75 (for all other fermentations). The schott flask was modified to accommodate a sample collection tube and a rubber stopper containing a cotton-plugged glass pipe to release CO_2_. Once the yeast starts fermenting, CO_2_ rapidly saturates the medium and soon an anaerobic environment is generated within the schott flask. Earlier studies suggested that the European Brewery Convention (EBC) tall tube fermentors successfully allow prediction of the performance of yeast strains in full-scale fermentors [31]. Our primary results showed highly comparable growth and fermentation performance of yeasts in the fermentation setup used in this project and in the EBC tall tube fermentors. Therefore, our fermentation setup should allow to reliably predict and evaluate the fermentation characteristics of yeast strains in industrial conditions.

Finally, the five most promising strains were selected for semi-anaerobic batch fermentations in lignocellulosic hydrolysates (Additional file 1: Table S1) using the same fermentation setup as above. Unsaccharified spruce hydrolysate slurry was kindly provided by the SEKAB E-Technology AB, (Örnsköldsvik, Sweden). The lignocellulosic hydrolysate was diluted to 60% w/w with Milli Q water before starting the saccharification and the pH was adjusted to 4.7. Subsequently, the hydrolysate was saccharified using cellulase complex NS50013 (Novozymes, Bagsværd, Denmark) and β-glucosidase NS50010 (Novozymes, Bagsværd, Denmark) following the recommended enzyme dosage and saccharification temperature (47 °C). Similar to the very-high-gravity fermentation conditions, the starting OD_600_ for the lignocellulosic hydrolysate fermentation was 4. All fermentations were performed in duplicate. The fermentations were continuously stirred at 120 rpm (300 rpm for hydrolysate) and incubated at 30 °C (very-high-gravity, 5-HMF stress, and acetic acid stress), 40 °C (heat stress), or 35 °C (Inhibitor cocktail stress and lignocellulosic hydrolysate). The weight loss of the flasks due to CO_2_ release was used to follow the course of fermentation. The fermentations were terminated when the CO_2_ production rate for most of the strains dropped below 0.01 g L^−1^ h^−1^ and a 1 mL sample from each fermentation was taken to measure final ethanol titer using high-performance liquid chromatography (HPLC, Waters Isocratic Breeze, ion exchange column WAT010290) [16]. Column temperature was maintained at 75 °C. Five millimolar H_2_SO_4_ was used as eluent with a flow rate of 1 ml min^−1^. A refractive index detector (Waters 2410, Waters, Milford, MA, USA) was used to quantify the compounds of interest. Fermentation data were analyzed using Prism 6.04, Graph Pad Software (San Diego, CA, USA). CO_2_ production rate (g L^−1^ h^−1^) was measured using the cubic spline fitting function and by calculating the first derivative of the curve.

## Results and discussion

### Osmotolerance of non-conventional yeasts

We successfully identified several non-conventional osmotolerant yeast species using agar plates with increasing concentrations of glucose, fructose, and sorbitol. For most yeast species, similar results were obtained on the three test media. Therefore, we discuss below mainly our results for glucose tolerance. Previously, we found that some *S. cerevisiae* can tolerate 50% w/v glucose [15]. The tolerance limits of all yeast species tested in this study are given in Table 2. Interestingly, at least some isolates of most of the evaluated yeast species tolerated ≥40% w/v glucose (64 out of 82 species evaluated). This is somewhat surprising as the tested strains of many of those species were isolated from environments that are devoid of high sugar concentrations (Additional file 2) and thus would appear as less likely to have developed osmotic stress-specific tolerance mechanisms. Therefore, this tolerance to high sugar could be attributed to high general stress tolerance of these species. It is well known that ability to efficiently transport glycerol into the cells is an essential mechanism to combat osmotic stress in many yeast species [32]. Therefore, it is also possible that most of these yeast species have a potent glycerol production pathway and uptake mechanism.

**Table 2:**
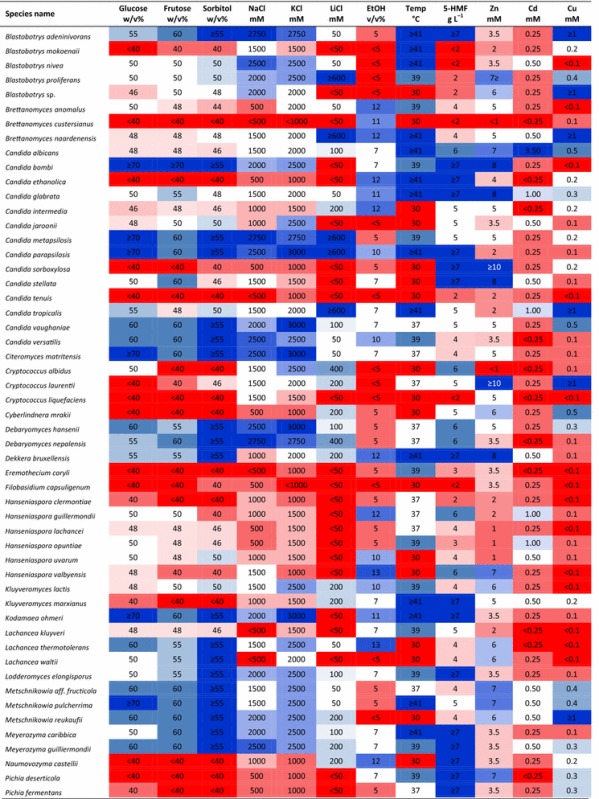

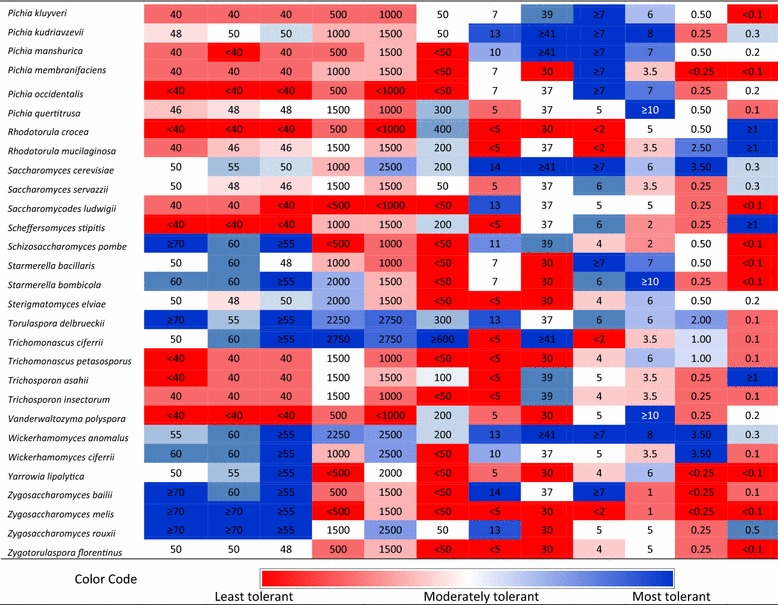
Summary of tolerance limits of various non-conventional yeast species observed under different stresses typically associated with second-generation bioethanol production

Tolerance limit = Most challenging test concentration for each species at which at least one isolate of that species showed RG ≥5%

In this study, 23 non-conventional yeast species showed significantly more osmotolerance than *S. cerevisiae*, and were able to grow on test plates with >55% w/v glucose. Moreover, strains from 11 species were able to grow on 70% w/v glucose, including *Candida bombi, Candida metapsilosis, Candida parapsilosis, Citeromyces matritensis, Kodamaea ohmeri, Metschnikowia pulcherrima, Schizosaccharomyces pombe, Torulaspora delbrueckii, Zygosaccharomyces bailii, Zygosaccharomyces mellis,* and *Zygosaccharomyces rouxii*. The majority of these osmotolerant strains were isolated from sugar-rich environments such as honey, maple syrup, beet sugar thick juice, molasses, and floral nectar and some were isolated from fermented cacao beans. As most of these species evolved independently from one another [33], it will be interesting to see whether or not they share the same genetic mechanisms to withstand high osmotic stress.

Among the most osmotolerant isolates, a *Z. rouxii* strain (isolated from Maple syrup) showed the highest relative growth (83%) under the reference condition (50% w/v glucose) followed by a strain of *C. matritensis* (isolated from beet sugar thick Juice) (66%) (Table 1). *Z. rouxii* is known for its superior osmotolerance [23, 34]. In this study, six out of seven *Z. rouxii* isolates managed to grow at 50% w/v glucose (Table 1). Although the molecular basis of the superior osmotolerance in *Z. rouxii* is not well understood, a number of possible mechanisms have been suggested. For example, two *Z. rouxii* plasma membrane sugar transporters, ZrFfz1 and ZrFfz2, with different substrate preferences (ZrFfz1 for fructose and ZrFfz2 for glucose) have been identified and are thought to play a role in the osmotolerance of this yeast [35]. In another study, the genes *ZrSTL1* and *ZrSTL2*, encoding transporters mediating active uptake of glycerol in symport with protons, have been linked with osmotolerance. Unlike *STL1* in *S. cerevisiae*, the *Z. rouxii STL* genes are not repressed by glucose. Therefore, these genes actively contribute to the maintenance of glycerol and intracellular pH homeostasis, which is essential for survival under hyperosmotic stress [36]. *C. matritensis* on the other hand has rarely been a research subject. All six isolates of *C. matritensis* evaluated in this study managed to grow at 50% w/v glucose (reference condition) (Table 1). Previously, this species has been found to be tolerant to high salt concentrations [37]. However, to the best of our knowledge this is the first time that this species is being reported for high sugar tolerance. Species such as *M. pulcherrima* and *C. bombi* have been found in flower nectar [38–41], which harbors sugar concentrations up to 50%. Yeast species such as *S. pombe* and *T. delbrueckii* are often associated with wine production. *S. pombe* is often described as a wine spoilage organism [42]. It has never been described before as an extremely osmotolerant species. Nevertheless, in this study, five out of six *S. pombe* isolates showed growth at 50% w/v glucose (reference condition) and, therefore, can be considered extremely osmotolerant. *T. delbrueckii* has been evaluated previously for high sugar grape must fermentations [43], rectification for stuck wine fermentations [44], and for brewing [45]. *T. delbrueckii* and *S. cerevisiae* are closely related based on sequence comparison [46–48]. Additionally, in some exceptional cases non-conventional yeasts showed better growth at the same concentration of fructose than of glucose, which could be an indication for a fructophilic character (Table 1). The most notable difference was observed for strains of *Z. rouxii* and *Wickerhamomyces anomalus*, which showed a relative growth of 102% and 150%, on 50% w/v fructose, respectively, whereas on 50% glucose this was 83 and 37%, respectively. This is in line with previous reports in which both of these species were described as fructophilic [35, 49]. Other noticeable differences were observed for strains of *T. delbrueckii, Trichomonascus ciferrii, Z. bailii*, and *Z. melis*.

### Halotolerance of non-conventional yeasts

In this study, three different salts (NaCl, KCl, and LiCl) were used to evaluate the yeasts’ halotolerance. Unlike osmotolerance, tolerance to the three types of salt was in most cases not comparable. In general, the isolates studied were most sensitive to LiCl, followed by NaCl and then KCl. A concentration of 50 mM LiCl was already sufficient to prevent growth of 39 out of the 82 species evaluated. On the other hand, even at 500 mM of NaCl, all species except 10 managed to grow and only seven species were inhibited by 1000 mM of KCl. A summary of the salt tolerance limits for all species investigated is given in Table 2. None of the isolates managed to grow on test plates containing 3000 mM NaCl. Strains of only four species managed to grow at a concentration of 2750 mM NaCl, including *Blastobotrys adeninivorans*, *Candida metapsilosis*, *Debaryomyces nepalensis*, and *T. ciferrii,* representing the most NaCl-tolerant species identified in this study. Yeast species such as *Blastobotrys nivea, Candida parapsilosis, Candida versatilis, C. matritensis*, and *Debaryomyces hansenii* tolerated 2500 mM of NaCl. Species that showed high NaCl tolerance also demonstrated considerable tolerance to KCl and grew on test plates containing 2500 mM KCl and above. The most KCl-tolerant strains managed to grow at 3000 mM KCl. They belong to the species *C. parapsilosis, Candida vaughaniae, C. matritensis, D. hansenii*, and *K. ohmeri*. Strains of six yeast species managed to grow on plates containing the highest concentration of LiCl tested (600 mM). These included the species *Blastobotrys proliferans, Brettanomyces naardenensis, C. metapsilosis, C. parapsilosis, Candida tropicalis,* and *T. ciferrii*. Out of all the species tested, only *C. metapsilosis, C. parapsilosis*, and *T. ciferrii* showed extreme tolerance to all three salts tested. This suggests that for most yeast species there is probably no common tolerance mechanism for different types of salts. It was also observed that several osmotolerant species such as *C. bombi, C. metapsilosis, C. parapsilosis, C. vaughaniae, C. versatilis, C. matritensis, K. ohmeri, Metschnikowia* sp., *Meyerozyma caribbica, Meyerozyma guilliermondii, T. delbrueckii*, and *W. anomalus* showed high tolerance under salt stress (Table 2), suggesting a common molecular mechanism to withstand sugar and salt stress. Indeed, for example, synthesis of polyols has been proposed as a possible mechanism used by both sugar- and salt-tolerant yeast strains [50]. However, in this study exceptions were also identified. For example, *S. pombe* and strains from the *Zygosaccharomyces* genus, which were identified as osmotolerant, were found to be sensitive to salt stress. This may suggest a dedicated osmotolerance mechanism of these species under high sugar concentrations, which is ineffective against ion toxicity due to salt stress. In order to learn more on different halotolerance mechanisms of yeasts, the review by Gunde-Cimerman and co-workers can be consulted [51]. Additionally, our results show that a few halotolerant species managed to grow better under reference conditions (NaCl 1500 mM and KCl 2000 mM) than on the control plate. The most remarkable difference was observed for strains belonging to *B. adeninivorans, D. hansenii, D. nepalensis*, and *W. anomalus*, which are extremely halotolerant (Table 1) and also appear to be halophilic [52]. In agreement to previous observations, our study identified *C. matritensis* [37] and *D. hansenii* [53] as extremely halotolerant species. *D. hansenii* is one of the most extensively studied halotolerant yeast species and has been frequently isolated from sea water [54] and other natural hypersaline environments [52].

### Ethanol tolerance of non-conventional yeasts

The majority of the yeast species tested managed to tolerate at least 5% v/v ethanol concentration (62 out of 82 tested species) while at 10% v/v ethanol, growth of 55 species was inhibited (no growth for any of the strains tested for these species). As expected, *S. cerevisiae* was the most ethanol-tolerant yeast species in our study, tolerating up to 14% v/v ethanol. However, several non-conventional yeast species were nearly as good as *S. cerevisiae*. Among all the yeast species evaluated, a *Z. bailii* strain (isolated from Orange wine, Congo) showed similar ethanol tolerance to *S. cerevisiae* (14% v/v). Moreover, the maximal ethanol tolerance of several yeast species such as *Hanseniaspora valbyensis, Lachancea thermotolerans, P. kudriavzevii, Saccharomycodes ludwigii, T. delbrueckii, W. anomalus,* and *Z. rouxii* was 13% v/v, which is only slightly lower than that of *S. cerevisiae*. While for most of these species no information was available on their ethanol tolerance, the high ethanol tolerance of *T. delbrueckii* was anticipated because of its direct association with wine production [43, 44, 55]. In our study, we also incorporated several *Brettanomyces* strains that are used in the brewing of specific beers, such as lambic beers [56, 57]. Species within this genus have been reported to display high ethanol tolerance and for appearing and even dominating in wine and industrial bioethanol fermentations as contaminant [58–60]. However, in our study most of the yeast species within this genus, such as *Brettanomyces anomalus, Brettanomyces (Dekkera) bruxellensis*, and *Brettanomyces naardenensis* did not grow on test plates containing more than 12% v/v ethanol. This suggests that very-high-gravity fermentation, in which a higher ethanol titer is reached, might be a suitable strategy to avoid such industrial contaminants. On the other hand, several *Brettanomyces* strains have also been reported as promising hosts for bioethanol production, especially for fermentation at low pH and also when the relative amount of nitrate can be high [61]. Interestingly, most of the ethanol-tolerant species identified in our study, namely *H. valbyensis, L. thermotolerans, S. ludwigii, S. cerevisiae, T. delbrueckii*, *Z. bailii*, and *Z. rouxii,* are genetically closely related to one another and all belong to the families *Saccharomycetaceae* and *Saccharomycodaceae* [48]. This raises the possibility of a common molecular event preceding the divergence of these species during the course of evolution that led to high ethanol tolerance. Other ethanol-tolerant species identified in our study, including the species *W. anomalus* and *Pichia kudriavzevii,* are well known to be generally robust micro-organisms due to their ability to thrive under a wide range of environmental conditions. *W. anomalus,* also known as *Pichia anomala,* is tolerant to several environmental stress factors such as a wide range of temperatures from 3 to 37 °C, pH values from 2 to 12, and hyperosmotic stress: water activity (a_w_) level of 0.85 [62–64]. However, this is the first time that its high ethanol tolerance has been reported. Similarly, *P. kudriavzevii,* also known as *Issatchenkia orientalis*, has been reported for its high tolerance to furan aldehydes [26, 28], high temperature [65, 66], extremely low pH conditions (down to pH 2) [67, 68], osmotic stress [28], and ethanol stress [68]. Therefore, the multi-stress tolerance of *W. anomalus* and *P. kudriavzevii* could be attributed to a very efficient general stress tolerance mechanism.

### Thermotolerance of non-conventional yeasts

For commercially viable second-generation bioethanol production, a yeast is preferred that can ferment above 40 °C in order to reduce cooling costs [69], to prevent bacterial contamination [70], and to reduce the optimal temperature difference between enzymatic hydrolysis (45–50 °C) and fermentation (30–37 °C) [71, 72]. In this study, we have identified several yeast species that grow at 41 °C. Nineteen out of the 82 species evaluated managed to grow at 41 °C. In contrast, only one out of the nine *S. cerevisiae* strains tested managed to grow (although weakly, RG: 21%) at 41 °C. Some species showed much better growth at 37 °C (reference condition) than at lower temperatures, which indicates a higher optimum temperature for these species than the reference temperature of 30 °C. In this regard, *M. pulcherrima* and *P. kudriavzevii* showed remarkable improvement in growth at 37 °C and the spot size increased 568% and 300% compared to the growth on the control plate (30 °C), respectively (Table 1). *M. pulcherrima* and *P. kudriavzevii* were the most thermotolerant yeast species identified in this study followed by *K. marxianus*. All seven *P. kudriavzevii* strains tested in this study managed to grow at 41 °C with relative growth ranging from 90 to 643%, confirming earlier findings. More particularly, *P. kudriavzevii* has often been identified as a thermotolerant, ethanologenic yeast species [26, 65, 66, 73] with the ability to produce ethanol up to 45 °C [73]. Likewise, *K. marxianus* is a well-known thermotolerant yeast species with the ability to grow up to 52 °C [74] and to produce ethanol at temperatures above 40 °C [69, 75, 76]. *M. pulcherrima*, on the other hand, has not yet been reported previously as a thermotolerant species.

### 5-Hydroxymethylfurfural tolerance of non-conventional yeasts

Although furfural is more toxic than 5-HMF at an equimolar concentration, the conversion of 5-HMF into less toxic compounds by yeast during fermentation is much slower than that of furfural and takes place only when furfural is completely reduced [77, 78]. It is also well known that furfural and 5-HMF share the same degradation pathways and have similar toxicity mechanisms in yeast. Therefore, we decided to consider 5-HMF tolerance of the yeast isolates as a strong basis for evaluation of furan aldehyde tolerance in general. Depending on the nature of the feedstock and the pretreatment method, lignocellulosic hydrolysates may contain up to 3.4 g L^−1^ of 5-HMF [17, 79]. This concentration is sufficient to inhibit the growth of several *S. cerevisiae* strains [15]. Therefore, exploration of non-conventional yeast strains for higher tolerance to 5-HMF may improve the bioethanol production process and/or lead to the identification of novel genetic tools for improving 5-HMF tolerance in second-generation bioethanol producing *S. cerevisiae* strains. In this regard, a limited number of studies have been performed so far to evaluate the tolerance of non-conventional yeast species to 5-HMF. Here, we have identified 25 yeast species that can tolerate to some extent more than 7 g L^−1^ of 5-HMF. Species such as *C. bombi, Candida stellata, Pichia fermentans, Saccharomyces servazzii, Starmerella bacillaris, W. anomalus*, and *Z. bailii* showed nearly no growth inhibition (RG > 90%) at 5 g L^−1^ 5-HMF (reference condition) (Table 1). Even at a concentration of 7 g L^−1^ 5-HMF species such as *C. bombi, Candida ethanolica, C. stellata, Pichia kluyveri, Pichia occidentalis, S. bacillaris*, and *Z. bailii* sustained at least 50% of the spot size of the control plate without 5-HMF. Six out of seven strains of *P. kudriavzevii,* which has previously been described as a furan aldehyde-tolerant non-conventional yeast species [28, 80], managed to grow at 7 g L^−1^ 5-HMF, albeit at a relative growth within the range of 8 to 39%. A well-known strategy to develop a furan aldehyde-tolerant *S. cerevisiae* strain is to enhance aldehyde reducing activity by overexpression of an NADH or NADPH-dependent alcohol dehydrogenase, aldehyde dehydrogenase, or methylglyoxal reductase isoenzyme [13, 81–85]. It remains to be investigated whether these extremely 5-HMF-tolerant non-conventional yeasts have similar detoxification mechanisms, mediated by genes orthologous to the corresponding *S. cerevisiae* genes. Additionally, our study provides novel opportunities for identifying other detoxification mechanisms by investigating the 5-HMF-tolerant species identified in our study in more detail.

### Heavy metal tolerance of non-conventional yeasts

Sometimes lignocellulosic feedstocks are contaminated with heavy metals. This imposes a new challenge for second-generation bioethanol production as it necessitates an efficiently fermenting microorganism that is impervious to the presence of toxic heavy metals. In this study, yeasts were screened for growth in the presence of increasing concentrations of Zinc (Zn), Cadmium (Cd), and Copper (Cd) salts. The prevalence of these heavy metals as contaminants of soil and ground water is common in several European countries and mainly attributed to human activities such as industrialization and intensive agriculture [86]. Heavy metal toxicity may induce morphological changes [87], aberrancy in physiological properties such as inhibition of enzyme production [88], denaturation of proteins and nucleic acids, and formation of hydroxyl radicals [89] in yeasts. On the other hand, it is well known that trace amounts of, for example, zinc are essential as micronutrients for normal growth, metabolism, and physiology of yeasts [90]. Our study also found that low amounts of Zn (1 mM) inhibit the growth of only a small number of yeast strains (15 out of 232) while a large number of isolates (77 out of 232) grew better than on the control plate. The most Zn-tolerant isolates identified in our study managed to grow at 10 mM Zn and belonged to the species *Candida sorboxylosa, Cryptococcus laurentii, Pichia quercitrusa, S. bombicola,* and *Vanderwaltozyma polyspora* (Table 2). Out of these, strains of *C. laurentii* and *V. polyspora* showed a better growth at the reference condition (5 mM) than on the control plates (Table 1). In the case of Cd tolerance, even at 0.25 mM the growth of 15 yeast species was inhibited (Table 2). The most tolerant yeast strains managed to grow at 3.5 mM and belonged to the species *C. albicans, S. cerevisiae, W. anomalus*, and *Wickerhamomyces ciferrii*. The strains of *W. anomalus* were most tolerant with a relative growth of 162%. Finally, 10 species managed to grow at 1 mM of Cu which is the highest concentration isolates were subjected to. *Trichosporon asahii* and *C. laurentii* showed exceptional Cu tolerance with relative growth of 322 and 263%, respectively. Other notable Cu-tolerant species were *Rhodotorula mucilaginosa, C. tropicalis, B. naardenensis, B. adeninivorans, Rhodotorula crocea,* and *M. reukaufii*. Interestingly, almost all the isolates that managed to grow in the presence of 1 mM Cu showed under this condition better growth compared to the control plate. Previously, only few studies have evaluated tolerance of yeast species to heavy metals [88, 91, 92]. For example, Vadkertiová and Sláviková [91] evaluated the tolerance of 15 yeast species, isolated from water, soil, and tree leaves, to four heavy metals, i.e., copper, zinc, nickel, and cadmium. In agreement with this study, our study also identified *C. laurentii* among the most Zn- and Cu-tolerant species. Little is known so far about the different mechanisms underlying metal tolerance in yeasts. On the other hand, a well-known tolerance mechanism of yeasts against Cu and Cd is the presence of multiple copies of the yeast metallothionein *CUP1* gene [93]. Cup1 binds to excess metal ions imported from the cellular environment. Li and co-workers also provided insight on the Cd tolerance mechanism of *S. cerevisiae*. Their study indicated that *S. cerevisiae* Ycf1, a member of the ATP-binding cassette (ABC) transporter family that is associated with multidrug resistance, pumps Cd conjugated to glutathione into vacuoles [94]. Currently, there is a growing interest to understand more about yeast adaptation to high concentrations of heavy metals in order to identify new tools for bioremediation of heavy metal-contaminated soils and water.

### Selection of multi-tolerant strains for small-scale semi-anaerobic batch fermentation assays

**Fig. 1:**
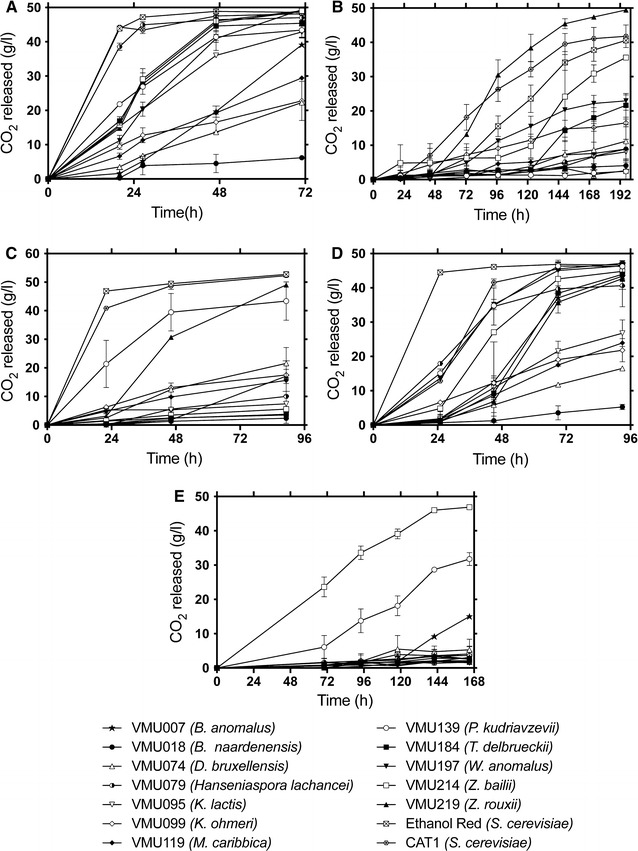
Fermentation efficiency of the selected multi-tolerant *Saccharomyces* (CAT1 and Ethanol Red) and non-*Saccharomyces* yeast strains [VMU007 (*B. anomalus*), VMU018 (*B. naardenensis*), VMU074 (*D. bruxellensis*), VMU079 (*H. lachancei*), VMU095 (*K. lactis*), VMU099 (*K. ohmeri*), VMU119 (*M. caribbica*), VMU139 (*P. kudriavzevii*), VMU184 (*T. delbrueckii*), VMU197 (*W. anomalus*), VMU214 (*Z. bailii*), and VMU219 (*Z. rouxii*)] in different stress conditions at 30 °C (unless mentioned otherwise). Fermentations were performed in duplicate. **a** glucose 10% (w/v) (control condition), **b** VHG condition (glucose 10% + sorbitol 50% w/v), **c** temperature 40 °C, **d** 3 g L^−1^ 5-HMF, and **e** 0.8% v/v acetic acid. Error bars represent the standard deviation from the mean

A summary is given of the most stress-tolerant yeast species identified in this study for all traits investigated (Table 3). In order to select yeast strains for the fermentation experiments, preference was given to strain performance in the presence of four commonly encountered stress factors in second-generation bioethanol fermentation, including osmotic stress, ethanol stress, 5-HMF stress, and heat stress. Furthermore, strain selection was kept limited to one strain per species. In this way, 12 strains that belong to 12 yeast species were selected for the fermentation experiments. More information on the strains, including taxonomic affiliation (species), tolerance limits to the stress factors, and percentage relative growth of the selected multi-tolerant strains at reference conditions is given in Table 4. First, all strains were subjected to fermentations at 30 °C that were devoid of any stress factors, i.e., YP 10% glucose (Fig. 1a). In this condition, all selected strains managed to start the fermentation but only the strains of *H. lachancei* (VMU079)*, K. lactis* (VMU095)*, P. kudriavzevii* (VMU139)*, T. delbrueckii* (VMU184)*, W. anomalus* (VMU197)*, Z. bailii* (VMU214), and *Z. rouxii* (VMU219) managed to virtually finish the fermentation yielding ethanol at more than 85% of the theoretical value (Table 5). On the other hand, the two industrial *S. cerevisiae* strains, included as a reference, yielded more than 90% of the theoretical value. None of the strains fermented faster than the *S. cerevisiae* strain CAT1. Among the non-*Saccharomyces* strains, *H. lachancei* (VMU079) and *Z. rouxii* (VMU219) were the fastest. Strains of *B. naardenensis* (VMU018)*, B. anomalus* (VMU007)*, D. bruxellensis* (VMU074)*, K. ohmeri* (VMU099), and *M. caribbica* (VMU119) had a poor fermentation profile with a lower Vmax (maximum rate of fermentation, g of CO_2_ released per liter hour) (Table 5) and longer lag phase as shown in Fig. 1a. *B. naardenensis* (VMU018) demonstrated the worst fermentation profile and accumulated only 7% of the theoretical ethanol yield. Further research is needed to find out why these strains were less effective in producing ethanol under the reference conditions used. Details of the fermentation profiles of each strain are presented in Table 5.

**Table 3:** Overview of the most stress-tolerant yeast species identified in this study

Osmotolerant	Halotolerant	Thermotolerant	Ethanol tolerant	HMF tolerant
*Blastobotrys adeninivorans*	*Blastobotrys adeninivorans*	*Blastobotrys adeninivorans*	*Hanseniaspora valbyensis*	*Blastobotrys adeninivorans*
*Candida bombi*	*Blastobotrys nivea*	*Blastobotrys mokoenaii*	*Lachancea thermotolerans*	*Candida bombi*
*Candida metapsilosis*	*Candida metapsilosis*	*Blastobotrys nivea*	*Pichia kudriavzevii*	*Candida ethanolica*
*Candida parapsilosis*	*Candida parapsilosis*	*Brettanomyces naardenensis*	*Saccharomyces cerevisiae*	*Candida glabrata*
*Candida vaughaniae*	*Candida vaughaniae*	*Candida albicans*	*Saccharomycodes ludwigii*	*Candida parapsilosis*
*Candida versatilis*	*Candida versatilis*	*Candida ethanolica*	*Torulaspora delbrueckii*	*Candida sorboxylosa*
*Citeromyces matritensis*	*Citeromyces matritensis*	*Candida glabrata*	*Wickerhamomyces anomalus*	*Candida stellata*
*Debaryomyces hansenii*	*Debaryomyces hansenii*	*Candida parapsilosis*	*Zygosaccharomyces bailii*	*Dekkera bruxellensis*
*Debaryomyces nepalensis*	*Debaryomyces nepalensis*	*Candida tropicalis*	*Zygosaccharomyces rouxii*	*Kluyveromyces marxianus*
*Kodamaea ohmeri*	*Kodamaea ohmeri*	*Dekkera bruxellensis*		*Kodamaea ohmeri*
*Lachancea thermotolerans*	*Meyerozyma guilliermondii*	*Kluyveromyces marxianus*		*Lodderomyces elongisporus*
*Metschnikowia aff. Fructicola*	*Torulaspora delbrueckii*	*Kodamaea ohmeri*		*Meyerozyma caribbica*
*Metschnikowia pulcherrima*	*Trichomonascus ciferrii*	*Metschnikowia pulcherrima*		*Meyerozyma guilliermondii*
*Metschnikowia reukaufii*	*Wickerhamomyces anomalus*	*Meyerozyma caribbica*		*Naumovozyma castellii*
*Meyerozyma guilliermondii*		*Pichia kudriavzevii*		*Pichia deserticola*
*Schizosaccharomyces pombe*		*Pichia manshurica*		*Pichia fermentans*
*Starmerella bombicola*		*Saccharomyces cerevisiae*		*Pichia kluyveri*
*Torulaspora delbrueckii*		*Trichomonascus ciferrii*		*Pichia kudriavzevii*
*Wickerhamomyces anomalus*		*Wickerhamomyces anomalus*		*Pichia manshurica*
*Wickerhamomyces ciferrii*				*Pichia membranifaciens*
*Zygosaccharomyces bailii*				*Pichia occidentalis*
*Zygosaccharomyces mellis*				*Saccharomyces cerevisiae*
*Zygosaccharomyces rouxii*				*Starmerella bacillaris*
				*Wickerhamomyces anomalus*
				*Zygosaccharomyces bailii*

Growth = Relative growth >5%, Osmotolerant (Growth on 60% w/v in glucose/fructose); Halotolerant (growth on 2500 mM NaCl/2750 mM KCl); Thermotolerant (growth >5% at 41 °C); ethanol tolerant (growth on 13% v/v ethanol); 5-HMF tolerant (growth on 7 g L^−1^ w/v 5-HMF)

**Table 4:** Overview of the tolerance limits and percentages relative growth (at reference conditions) of the selected multi-tolerant strains for the fermentation experiments

Strain	Species	Osmotolerance		Ethanol tolerance		HMF tolerance		Thermotolerance	
		Tolerance limit (w/v %)	Relative growth at 50% glucose (%)	Tolerance limit (v/v %)	Relative growth at 7% EtOH (%)	Tolerance limit (g L^−1^)	Relative growth at 5 g L^−1^ HMF (%)	Tolerance limit (°C)	Relative growth at 39 °C (%)
VMU007	*Brettanomyces anomalus*	50	8	11	138	4	0	39	183
VMU018	*Brettanomyces naardenensis*	48	2	7	35	4	2	>41	120
VMU074	*Dekkera bruxellensis*	50	12	7	36	>7	41	>41	77
VMU079	*Hanseniaspora lachancei*	50	7	12	74	6	70	39	5
VMU095	*Kluyveromyces lactis*	48	6	7	19	4	0	37	0
VMU099	*Kodamaea ohmeri*	>70	37	11	77	>7	77	>41	116
VMU119	*Meyerozyma caribbica*	50	17	7	38	>7	59	39	15
VMU139	*Pichia kudriavzevii*	48	1	13	120	>7	57	>41	491
VMU184	*Torulaspora delbrueckii*	55	13	11	47	6	22	37	0
VMU197	*Wickerhamomyces anomalus*	50	25	11	116	5	28	37	0
VMU214	*Zygosaccharomyces bailii*	>70	23	10	59	>7	96	37	0
VMU219	*Zygosaccharomyces rouxii*	>70	69	7	28	4	0	37	0
Ethanol Red	*Saccharomyces cerevisiae*	50	15	14	102	5	33	39	150
CAT1	*Saccharomyces cerevisiae*	50	20	13	71	2	0	39	41

**Table 5:** Performance comparison of the selected multi-tolerant strains in different fermentation conditions

Strain	Glucose 10% (w/v)				VHG Glu 10% + Sor 50% (w/v)				5-HMF 3 g L^−1^				Acetic acid 0.8% (v/v)				Temperature 40 °C			
	A	B	C	D	A	B	C	D	A	B	C	D	A	B	C	D	A	B	C	D
VMU007	73	0.83	70.3	53.6	19	0	∝	ng	93	1.13	55	56.4	56	0.35	134	>165	28	0.45	88.3	>89.2
VMU018	7	0.58	22.5	>71	17	0	∝	ng	6	0.11	59.7	>93	13	0	∝	ng	8	0	∝	ng
VMU074	47	0.51	23.9	>71	33	0.15	132	>196	36	0.26	43.7	>93	19	0.18	103	>165	54	0.45	37.3	>89.2
VMU079	90	2.24	0.72	11.6	28	0.12	140	>196	89	0.92	30.5	31.9	16	0	∝	ng	38	0.27	37.3	>89.2
VMU095	86	0.83	31.2	31.87	30	0.13	160	>196	47	0.52	42.7	79.9	13	0	∝	ng	18	0.3	0.91	>89.2
VMU099	45	0.52	21	>71	49	0.14	130	>196	49	0.31	49.3	>93	21	0	∝	ng	50	0.3	26.4	>89.2
VMU119	57	0.73	23.9	60.9	22	0.19	84.4	>196	43	0.41	42.7	93	15	0	∝	ng	35	0.22	0.91	>89.2
VMU139	100	1.21	0.72	23.2	15	0	∝	ng	98	1.04	34.3	34.8	73	0.49	129	132	101	1	0.91	25.5
VMU184	98	1.68	23.9	24.6	62	0.49	136	>196	95	1.37	56.8	57.3	14	0	∝	ng	15	0	∝	ng
VMU197	91	1.45	23.9	29	59	0.28	84.4	>196	97	1.16	34.3	34.8	12	0	∝	ng	10	0	∝	ng
VMU214	91	2.1	23.9	24.6	84	0.6	138	149	100	1.22	37.1	42.3	105	0.45	75.8	71.7	13	0	∝	ng
VMU219	85	2.15	23.9	24.6	105	0.76	80.4	87.3	89	1.37	57.8	59.2	19	0	∝	ng	106	1.29	37.3	40.1
Ethanol Red	90	2.81	0.72	8.69	91	0.44	132	125	100	2.28	0.47	11.3	17	0.12	103	>165	108	2.63	0.91	9.1
CAT1	93	3.12	0.72	8.69	102	0.4	58.6	91.3	100	1.61	33.3	32.9	19	0	∝	ng	107	2.22	0.91	10.9

Fermentations were carried out at 30 °C, unless mentioned otherwise

A = Theoretical ethanol yield (%) reached when fermentation stopped

B = Vmax, maximum rate of fermentation (g L^−1^ h)

C = Time to reach Vmax (h)

D = C50, time to consume 50% of the initial sugar content (h)

*ng* no growth

As a next step, the selected strains were subjected to fermentations in the presence of different stress factors relevant to second-generation bioethanol production. Strains were subjected to one stress factor at a time in order to better understand how the stress factors individually impact the fermentation profile. First, very-high-gravity fermentation under high osmostress (YP Glucose 10% Sorbitol 50%) clearly showed the remarkable potential of *Z. rouxii* (VMU219) for the production of bioethanol under osmostress (Fig. 1b). More particularly, this strain not only produced the highest ethanol titer but also finished the fermentation faster than the *S. cerevisiae* reference strains. The tested strain from *Z. bailii* (VMU214) was the second-best performing strain among the non-*Saccharomyces* strains but fermented much slower than the *Z. rouxii* and *S. cerevisiae* strains.

Next, the fermentation potential of the selected strains under heat stress (40 °C) was investigated (Fig. 1c). In agreement with previous reports [26, 65, 66, 73], *Pichia kudriavzevii* (VMU139) showed the best fermentation performance compared to the other non-conventional yeast strains but it was outperformed by the *S. cerevisiae* strains. Especially, the fermentation profile of Ethanol Red at 40 °C was nearly as good as for the control condition (30 °C). *P. kudriavzevii* is well known for its ability to ferment up to 45 °C [73]. Therefore, it is highly probable that at higher temperature *P. kudriavzevii* will outperform *S. cerevisiae*.

Next, fermentation under furan aldehyde stress was evaluated (Fig. 1d). Once again, the fermentation of the *S. cerevisiae* strain Ethanol Red was nearly unaffected by 3 g L^−1^ of 5-HMF, while the *S. cerevisiae* strain CAT1 appeared more sensitive to 5-HMF stress. Several non-*Saccharomyces* strains were as good as CAT1, including the tested strains belonging to *H. lachancei* (VMU079) (89% of theoretical yield), *P. kudriavzevii* (VMU139) (98% of theoretical yield), and *W. anomalus* (VMU197) (97% of theoretical yield). *T. delbrueckii* (VMU184)*, Z. bailii* (VMU214) and *Z. rouxii* (VMU219) also managed to nearly finish the fermentation and produced more than 89% of the theoretical ethanol yield. Identification of *P. kudriavzevii* as one of the best performing species under furan aldehyde stress is in alignment with previous reports and the results obtained in our phenotypic screening (see above*)*.

During pretreatment of lignocellulosic biomass, acetic acid is the most abundant weak acid generated, with a concentration ranging between 5 and 10 g L^−1^ [95–98]. It is produced when the hemicellulose acetyl groups are released during pretreatment. Tolerance to weak acids was not evaluated in our high-throughput phenotyping. However, all selected strains were subjected to fermentation with 0.8% v/v acetic acid. Under this condition, only the strain of *Zygosaccharomyces bailii* (VMU214) managed to finish the fermentation with an apparent 105% of the theoretical yield (Fig. 1e; Table 5). Apart from *Z. bailii, P. kudriavzevii* (VMU139) and *B. anomalus* (VMU007) showed a comparatively better fermentation profile than the other non-conventional yeast strains tested and accumulated 73 and 56% of the theoretical ethanol yield, respectively. All the other strains, including the two industrial strains of *S. cerevisiae*, had a very poor fermentation performance with accumulation of less than 20% of the theoretical ethanol yield. Both *Z. bailli* and *P. kudriavzevii* are known for acetic acid tolerance and ability to grow under low pH conditions [68, 73, 99]. The acetic acid tolerance mechanism of *Z. bailii* is yet to be elucidated. Our study indicates the importance of investigating this mechanism so that this information could be extrapolated for engineering acetic acid-tolerant *S. cerevisiae* strains.

**Fig. 2:**
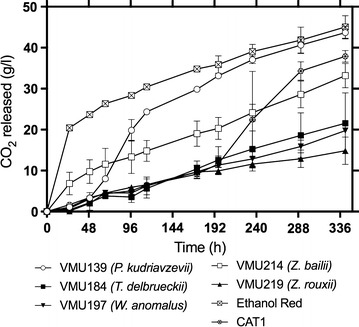
Lignocellulosic hydrolysate fermentation efficiency of selected multi-tolerant *Saccharomyces* (CAT1 and Ethanol Red) and non-S*accharomyces* yeast strains [VMU139 (*P. kudriavzevii*), VMU184 (*T. delbrueckii*), VMU197 (*W. anomalus*), VMU214 (*Z. bailii*), and VMU219 (*Z. rouxii*)]. Fermentations were performed in duplicate at 35 °C. Error bars represent the standard deviation from the mean

Finally, a fermentation experiment was performed for the five strains performing best in the previous assays using lignocellulosic hydrolysate. Results indicate that Ethanol Red was the best performing strain in such inhibitory conditions producing the highest ethanol yield (27.2 g L^−1^) and Vmax, i.e., the maximum rate of fermentation (0.97 g L^−1^ h) (Table 6; Fig. 2). However, *Pichia kudriavzevii* (VMU139) produced nearly the same ethanol yield (25.53 g L^−1^) compared to the industrial *S. cerevisiae* strain CAT1 (25.95 g L^−1^) and very close to that of the best performing *S. cerevisiae* strain Ethanol Red (27.20 g L^−1^) (Table 6) and it outperformed CAT1 in terms of Vmax (0.42 g L^−1^ h of VMU139 compared to 0.31 g L^−1^ h of CAT1). *P. kudriavzevii* (VMU139) was also much faster to reach the Vmax (81 h compared to 226 h taken by CAT1) (Fig. 2; Table 6). This shows that specific wild non-conventional yeast species have attractive capacities compared to highly evolved and selected commercial bioethanol *S. cerevisiae* strains when used in second-generation bioethanol fermentations. In addition, *P. kudriavzevii* is able to ferment at 45 °C [73], a temperature at which *S. cerevisiae* is unable to grow or ferment. Other than *P. kudriavzevii, W. anomalus* also managed to start the fermentation but resulted in a much lower ethanol yield (14.17 g L^−1^) than the *P. kudriavzevii* and *S. cerevisiae* strains (Table 6).

**Table 6:** Spruce hydroysate fermentation performance of selected multi-tolerant *Saccharomyces* and non-*Saccharomyces* yeast strains

Strain	Species	Spruce hydrolysate fermentation		
		A	B	C
VMU139	*Pichia kudriavzevii*	25.53	0.42	81
VMU184	*Torulaspora delbrueckii*	0.46	0.14	112
VMU197	*Wickerhamomyces anomalus*	14.17	0.30	2
VMU214	*Zygosaccharomyces bailii*	0.51	0.15	57
VMU219	*Zygosaccharomyces rouxii*	0.49	0.11	43
Ethanol Red	*Saccharomyces cerevisiae*	27.20	0.97	2
CAT1	*Saccharomyces cerevisiae*	25.95	0.31	226

A = Ethanol yield (g L^−1^) when fermentation stopped

B = Vmax, maximum rate of fermentation (g L^−1^ h)

C = Time to reach Vmax (h)

## Conclusions

This study illustrates the huge phenotypic variability among yeast species for tolerance to different stress factors relevant in second-generation bioethanol production. It is clear from our results that several non-conventional yeast species have attractive phenotypes that could be industrially exploited for second-generation bioethanol production. We identified several previously unreported non-conventional yeast species of which growth is highly tolerant to one or more of such stress factors. Additionally, our results show the potential of some non-conventional yeast species for fermentation under stressful conditions that occur in second-generation bioethanol production. We identified that a wild *Pichia kudriavzevii* (VMU139) strain can outperform the currently used industrial *S. cerevisiae* bioethanol strain CAT1 in second-generation hydrolysate fermentation. While Ethanol Red was the best performer in most of the fermentation conditions employed, its acetic acid sensitivity compromises its potential for second-generation bioethanol production. In this regard, our work reveals the potential of non-conventional yeast species such as *Pichia kudriavzevii* for lignocellulose hydrolysate fermentations due to their ability to produce ethanol even at high temperature and in the presence of high levels of acetic acid. On the other hand, this large-scale and high-throughput phenotypic survey has yielded a database on stress tolerance characteristics of non-conventional yeast species relevant in second-generation bioethanol production that can be used to select specific species for elucidation of the underlying stress tolerance mechanisms and transfer of the causative genes to industrial *S. cerevisiae* strains for second-generation bioethanol production. Moreover, it indicates excellent candidate non-*Saccharomyces* strains for evolutionary engineering, random and/or directed mutagenesis, whole-genome transformation, and other strain development methodologies in order to evaluate the limits of stress tolerance that can be reached in these non-conventional yeast species as opposed to what can be obtained in industrial *S. cerevisiae* strains.

## Additional files

**Additional file 1: Table S1.** Composition of lignocelluloses hydrolysate which was used for the final fermentation experiment.

**Additional file 2: Table S2. ** Source, geographical origin and details of stress tolerance characteristics of yeast strains used in this study.
