# Supplementary material for: Phenotypic landscape of non-conventional yeast species for different stress tolerance traits desirable in bioethanol fermentation
Source: Biotechnol Biofuels. 2017 Sep 13;10:216. doi: 10.1186/s13068-017-0899-5 (PMC5597992; doi:10.1186/s13068-017-0899-5)
Supplement: Supplementary file 1 — Additional file 1: Table S1. Composition of lignocelluloses hydrolysate which was used for the final fermentation experiment. [file 13068_2017_899_MOESM1_ESM.docx]

**Table S1**: Composition of lignocelluloses hydrolysate which was used for the final fermentation experiment.

| **Composition of the spruce hydrolysate [Liquid g/l (oligomer g/l), solid g/kg]** | | |
| --- | --- | --- |
| Pretreatment | pH 2,0 18 bar 203◦C 5-6 min | pH 2,0 18 bar 203◦C 5-6 min |
| Phase | Liquid | Solid |
| Glucose | 42 (2,89) | 468 |
| Mannose | 29 (2,7) | 2.64 |
| Arabinose | 3.2 | 0.21 |
| Formic acid | 0.3 |  |
| Acetic acid | 7 |  |
| Levulinic acid | 1.7 |  |
| Xylose | 13 | 2.14 |
| Galactose | 5,5 (0,61) | 0.25 |
| 5-HMF | 5.3 |  |
| Furfural | 2.7 |  |
| Lignin |  | 49.7 |
| %Total Solid in hydrolysate=27.6 | |  |
